# Supplementary material for: Four Cycles of Docetaxel-Cyclophosphamide versus Anthracycline-Taxane as Adjuvant Chemotherapy for HER2-Negative, Axillary Lymph Node Negative Breast Cancer: A Real-World Comparison of Alberta Patients Treated 2008–2012
Source: Curr Oncol. 2021 Mar 4;28(2):1137–42. doi: 10.3390/curroncol28020109 (PMC8025747; doi:10.3390/curroncol28020109)
Supplement: Supplementary file 1 [file curroncol-28-00109-s001.pdf]

Current Oncology

Supplementary Materials

Four Cycles of Docetaxel-Cyclophosphamide Versus Anthracycline-Taxaneas Adjuvant Chemotherapy for HER2-Negative, Axillary Lymph Node Negative Breast Cancer: A Real-World Comparison of Alberta Patients Treated 2008-2012

Malek Hannouf [1], Atul Batra [2,3] and Sasha Lupichuk [2,*]

Table S1. Cox regression analyses for iDFS, BCSS, and OS.

**Proportional-hazard models***		**Patients with DC4 vs. patients with AT**								
		**iDFS**			**BCSS**			**OS**		
		**HR**	**95% CI**	***p*-value**	**HR**	**95% CI**	***p*-value**	**HR**	**95% CI**	***p*-value**
Matched patients with DC4 vs. patients with AT (1:4 matching; n = 271)		1.05	0.65 - 1.8	0.85	0.91	0.42 - 1.9	0.8	0.98	0.46 - 2.1	0.96
All patients with DC4 vs. patients with AT (n = 726)	Unadjusted for patient and disease characteristics†	0.87	0.52 - 1.5	0.61	0.73	0.36 - 1.4	0.4	0.94	0.47 - 1.8	0.88
	Adjusted for patient and disease characteristics†	0.98	0.56 - 1.7	0.94	0.96	0.46 - 1.9	0.9	0.92	0.44 - 1.9	0.83
	Adjusted for the estimated propensity score	0.92	0.51 - 1.6	0.77	0.92	0.46 - 1.9	0.9	0.96	0.55 - 1.7	0.92
	Adjusted for patient and disease characteristics using inverse probability weighting by the inverse of the propensity score	1.06	0.57 - 1.9	0.84	0.90	0.57 - 1.9	1.0	0.95	0.6 - 1.7	0.94

*We created time dependent covariates by creating interactions of the covariates and a function of survival time in the models to test proportionality. When time dependent covariates were not significant then covariates were considered proportional. The proportionality assumption was appropriate for all. † Patient and disease characteristics included age, sex, co-morbidity, number and type of metastatic sites, grade differentiation, histology, and year of initial diagnosis. Abbreviations: HR = Hazard ratio; iDFS = Invasive disease free survival; BCSS = Breast cancer specific-survival; OS =. Overall survival; DC×4 = Four cycles of docetaxel-cyclophosphamide; AT= Anthracycline-taxane regimen.
